# Mitochondrial DNA haplogroups in early-onset Alzheimer's disease and frontotemporal lobar degeneration

**DOI:** 10.1186/1750-1326-5-8

**Published:** 2010-02-02

**Authors:** Johanna Krüger, Reetta Hinttala, Kari Majamaa, Anne M Remes

**Affiliations:** 1 Institute of Clinical Medicine, Neurology, University of Oulu, Oulu, Finland; 2 Clinical Research Center, Oulu University Hospital, Oulu, Finland

## Abstract

**Background:**

Mitochondrial dysfunction, oxidative damage and the accumulation of somatic mutations in mitochondrial DNA (mtDNA) have been associated with certain neurodegenerative disorders. Previous studies have also provided controversial results on the association of mtDNA haplogroups with susceptibility to Alzheimer's disease (AD), but possible relationships between mtDNA and frontotemporal lobar degeneration (FTLD) have been less frequently studied.

**Methods:**

We analysed the role of mtDNA and its maintenance enzymes in 128 early-onset AD (eoAD) and in 66 FTLD cases. Patients and 99 controls were collected from a defined region of Finland, that of Northern Ostrobothnia, for the determination of mtDNA haplogroups and the analysis of two common mtDNA mutations (m.3243A>G, m.8344A>G). In addition, screening was performed for five common *POLG1 *mutations (T251I, A467T, P587L, W748S and Y955C) and all the coding exons of the *PEO1 *and *ANT1 *genes were screened for mutations.

**Results:**

The frequency of haplogroup cluster IWX was 2.3 fold higher among the FTLD cases than in the controls (OR 2.69, 95% CI 1.09-6.65, *p *= 0.028). The frequency of mtDNA haplogroups or clusters did not differ between the eoAD cases and controls. The two mtDNA mutations and five *POLG1 *mutations were absent in the eoAD and FTLD patients. No pathogenic mutations were found in the *PEO1 *or *ANT1 *genes.

**Conclusions:**

We conclude that the haplogroup cluster IWX was associated with FTLD in our cohort. Further studies in other ethnically distinct cohorts are needed to clarify the contribution of mtDNA haplogroups to FTLD and AD.

## Background

Alzheimer's disease (AD) and frontotemporal lobar degeneration (FTLD) are the two most common neurodegenerative diseases leading to early-onset dementia. Causative mutations in the presenilin 1 (*PSEN1*), presenilin 2 (*PSEN2*) and amyloid precursor protein (*APP*) genes have been found in a few patients with familial AD [1]. The ε4 allele of the apolipoprotein E gene (ApoE) has a significant association with AD [2]. Pathogenic mutations in the microtubule-associated protein tau (*MAPT*) and progranulin (*PGRN*) genes have rarely been identified in FTLD despite the high frequency of familial cases [3-5]. Most AD and FTLD cases have a complex etiology, however, and many genetic and also behavioural and environmental risk factors may play a role in the disease processes.

There is growing evidence to suggest that mitochondria play an important role in ageing and in neurodegenerative diseases. Mitochondrial dysfunction leads to reduced ATP production, increased generation of reactive oxygen species and also impaired calcium buffering [6]. The brain is especially vulnerable to oxidative damage, and mutations in mitochondrial DNA (mtDNA) and in the genes involved in mtDNA maintenance are associated with variable spectrum of mitochondrial disorders affecting the brain [7,8]. *POLG1*, encoding the catalytic subunit of DNA polymerase γ, *PEO1*, coding for the mitochondrial helicase Twinkle, and *ANT1*, encoding the adenine nucleotide translocator, are important for mtDNA replication. Pathogenic mutations in mtDNA or in its maintenance genes have rarely been described in neurodegenerative diseases leading to dementia. Y955C mutation in *POLG1 *was previously identified in a patient with progressive external opthalmoplegia (PEO) who had Alzheimer pathology and multiple mtDNA deletions [9], There are also some patients with pathogenic mtDNA mutations suffering from dementia and cognitive impairment [10]. MtDNA polymorphisms and certain haplogroups have been associated with neurodegenerative diseases such as Parkinson's disease and AD [11-13] and also with longevity [14-16]. Previous studies have reached variable conclusions with regard to the association of certain mtDNA haplogroups with AD. Men with haplogroup U had an increased risk of AD, while women with the same haplogroup had a significant decrease in that risk [17]. Furthermore, haplogroups K and U seemed to neutralize the effect of the ε4 allele of ApoE [18]. However, in some studies no association was found between mtDNA haplogroups and AD [19-21]. So far there is only one report on frontotemporal dementia and mtDNA haplogroups, reporting no evidence of any association between them [22].

We report here on investigations into the role of mtDNA and its maintenance enzymes in AD and FTLD in Finnish patients. Patients and controls were collected from a defined region of Finland, that of Northern Ostrobothnia, for the determination of mtDNA haplogroups and the analysis of two common mtDNA mutations (m.3243A>G, m.8344A>G) in the patients. Since encephalopathy and cognitive decline are common features in mitochondrial diseases, screening was performed for five common *POLG1 *mutations (T251I, A467T, P587L, W748S and Y955C) and all the coding exons of the *PEO1 *and *ANT1 *genes were screened for mutations.

## Patients and methods

### Subjects

We examined 194 patients (92 men) with early-onset AD and FTLD at the Department of Neurology of Oulu University Hospital, Finland. The patients were all from the population of the province of Northern Ostrobothnia and their parents were all of Finnish origin. Patients from other ethnic groups were not able to participate the study. Probable AD had been diagnosed according to the NINCDS-ADRDA criteria [23] in 128 cases and FTLD according to the clinical diagnostic criteria for that disease in 66 cases [24]. The mean age at onset was 58 years (range 38-64) for the AD patients and 58.5 years (range 40-79) for the FTLD patients.

Use was made of results obtained previously from a mtDNA haplogroup analysis of 99 control subjects (mean age 40 ± 12 years, range 19-64) from Northern Ostrobotnia [25]. The controls were healthy anonymous blood donors whose mothers had also been free of neurological diseases and the common manifestations of mitochondrial diseases such as hearing impairment and diabetes mellitus. Furthermore, the donors and their mothers were required to be born in the same province, in order to obtain a good representation of the ancestral population of the province.

Written informed consent was obtained from all the individuals or those caring for them. The research protocols were approved by the Ethics Committees of the Northern Ostrobothnia Hospital District and the Finnish Red Cross.

### Molecular methods

Total genomic DNA was extracted from the blood samples by the standard sodium dodecyl sulphate-proteinase K method. Fragments amplified by the polymerase chain reaction (PCR) technique were used for restriction fragment analysis (RFLP), conformation-sensitive gel electrophoresis (CSGE) [26], sequencing and mtDNA haplogroup analysis.

The m.3243A>G mutation was screened by RFLP using the restriction enzyme *ApaI *[27]. The m.8344A>G mutation was detected by RFLP using *BglI *[28]. MtDNA haplogroups (H, V, U, K, J, T, I, W, X) were determined by RFLP of polymorphic sites as described previously [29,30]. These haplogroups can be grouped into clusters (HV, UK, JT, IWX) according to their phylogenetic background [30].

The *POLG1 *mutations T251I (*BseNI*), A467T (*MscI*) and P587L (*XmaI*) were analysed by RFLP, while the W748S mutation was verified by allele-specific amplification using primers with a locked nucleic acid (LNA) nucleoside base at the 3'end (Proligo LLC, Paris, France). The primers were designed to anneal with either the wild type sequence (5'-GGACATCCCTGGCTGCT**G**-3') or the sequence containing the mutation (5'-GGACATCCCTGGCTGCT**C**-3'). Detection of the Y955C mutation was performed by CSGE. The POLG1 exon 18, coding for the Y955, was amplified from the patient samples and from a control sample with a known sequence. The amplified fragments were mixed, denatured at 95°C for 5 min and the heteroduplexes were allowed to anneal at 68°C for 30 min. The samples were electrophoresed through a polyacrylamide gel overnight at a constant voltage of 400 V, stained in 10 μl/100 ml SYBR Gold solution (Invitrogen, Eugene, Oregon, U.S.A.) for 5 min, visualized with an UV transluminator and photographed (Chemi-Doc XRS, Bio-Rad Laboratories, CA, U.S.A.).

All the coding exons and exon-intron boundaries of *PEO1 *and *ANT1 *were screened using CSGE. Fragments were further sequenced if they differed in mobility on the CSGE. Sequencing was carried out using the BigDye Terminator v1.1 Cycle Sequencing Kit (Applied Biosystems, Foster City, CA, U.S.A.) and the ABI PRISM 3100 Genetic Analyzer (Applied Biosystems). The apolipoprotein E genotype was determined as described previously [31].

### Statistical analyses

Differences in the frequencies of mtDNA haplogroups and haplogroup clusters between the populations were evaluated using the exact test of population differentiation [32] as implemented in Arlequin 2.0 or, if applicable, using the χ^2 ^test or Fisher's exact test in the SPSS 16.0 software for Windows. The referent group consisted of all cases classified as carriers of other haplogroup or haplogroup cluster than the one under study. The minimum significance level was set at two-sided *p *= 0.05. The 95% confidence intervals of odds ratio (OR) were calculated. The patients and controls were stratified into two subgroups according to their ApoE genotype: individuals carrying at least one ApoE ε4 allele (ApoE4+) and non-ApoE ε4 carriers (ApoE4-).

## Results

MtDNA haplogroups were determined in 128 patients with eoAD and 66 with FTLD and compared with the previous results for the 99 controls (Table 1). The haplogroup frequencies differed between the three groups (*p *= 0.04; exact test of population differentiation). No significant difference was found in the frequency of haplogroups when patients with eoAD and patients with FTLD were separately compared to the controls (*p *= 0.41 and *p *= 0.074, respectively, exact test of population differentiation). However, a difference was found between patients with eoAD and those with FTLD (*p *= 0.006).

**Table 1:** Frequencies of mtDNA haplogroups among the eoAD and FTLD patients and the controls.

	eoAD		FTLD		Controls	
Haplogroup	N	%	n	%	n	%
H	56	43.8	29	43.9	34	34.3
I	0	0	2	3.0	0	0.0
J	7	5.5	2	3.0	2	2.0
K	5	3.9	2	3.0	3	3.0
T	7	5.5	3	4.5	8	8.1
U	41	32.0	15	22.7	34	34.3
V	6	4.7	0	0	6	6.1
W	5	3.9	9	13.6	8	8.1
X	0	0	3	4.5	1	1.0
Other	1	0.8	1	1.5	3	3.1
Total	128	100.1	66	99.7	99	100

Subsequently, we analysed the frequencies of clusters of phylogenetically related haplogroups HV, UK, JT and IWX (Table 2). The frequency of haplogroup cluster IWX was 2.3 fold higher among the FTLD cases than in the controls (OR 2.69, 95% CI 1.09-6.65, *p *= 0.028). No significant differences were observed in the frequencies of haplogroup clusters HV, UK and JT between the FTLD cases and controls (Table 3). The frequency of haplogroup W was higher among the patients with FTLD than in the controls but not significantly so (*p *= 0.25).

**Table 2:** Frequencies of mtDNA haplogroup clusters among the eoAD and FTLD patients and the controls.

Cluster	Cluster frequency							
	
	All patients		eoAD		FTLD		Controls	
	
	n	%	n	%	n	%	n	%
HV	91	46.9	62	48.4	29	43.9	40	40.4
IWX	19	9.8	5	3.9	14	21.2	9	9.1
JT	19	9.8	14	10.9	5	7.6	10	10.1
UK	63	32.4	46	35.9	17	25.7	37	37.4
Other	2	1.0	1	0.8	1	1.5	3	3.0
Total	194	99.9	128	99.9	66	99.9	99	100

**Table 3:** Odds ratios of mtDNA haplogroup clusters by comparison with referent category consisting of all cases classified as carriers of other clusters than the one under study.

Cluster	OR	95% CI	*p*-value
**eoAD compared with controls**			
HV	1.39	0.82 - 2.36	0.23
IWX	0.41	0.13 - 1.25	0.11
JT	1.09	0.46 - 2.58	0.65
UK	0.94	0.55 - 1.62	0.82
**FTLD compared with controls**			
HV	1.16	0.62 - 2.17	0.65
IWX	2.69	1.09 - 6.65	0.028
JT	0.73	0.24 - 2.24	0.73
UK	0.58	0.29 - 1.15	0.12

The frequencies of mtDNA haplogroups were not significantly different between the eoAD patients and the controls (Table 1). Haplogroup H was the most common in both the control and patient groups (Table 1). The frequency of haplogroup H was slightly but not significantly higher in the patients with eoAD than in the controls (*p *= 0.15). The frequency of cluster HV was higher, but not significantly so, in the AD patients than that in the controls (*p *= 0.23). ApoE4 allele was more frequent in AD patients than in controls (63.3% vs 35.1%, respectively). We found no association between carriers of ApoE4 allele and mtDNA haplogroups.

In addition, we analysed two mtDNA mutations and mutations in three nuclear-encoded enzymes which contribute to mtDNA integrity. The m.3243A>G and m.8344A>G mutations and the five common *POLG1 *mutations, T251I, A467T, P587L, W748S and Y955C, were absent in this population. Four single nucleotide polymorphisms (SNP) and one single nucleotide insertion were found in the *PEO1 *and *ANT1 *genes, but no pathogenic mutations (see additional files 1 and 2). One of the polymorphisms has not been reported previously.

## Discussion

We found a significant association between haplogroup cluster IWX and FTLD. Within this cluster, frequency of haplogroup W was highest in FTLD patients compared to the controls and eoAD patients. Interestingly, the non-synonymous/synonymous rate in the mtDNA-encoded complex I genes (MTND) is higher within haplogroup cluster IWX than in the remaining European haplogroup clusters [12] thus suggesting that the relative excess of nonsynonymous mutations in cluster IWX may have a role in the risk of developing FTLD. Another possible explanation for the observed association found in our cohort between cluster IWX and FTLD is a recent founder effect. There have been only a few previous reports of associations between the haplogroup W and certain diseases mainly due to its presence at low level in the population making it difficult to detect associations. In one report, however, an association is demonstrated for Iranian Leber hereditary optic neuropathy (LHON) patients with the m.3460G>A mutation, suggesting that W might be a haplotype that increases the penetrance of LHON [33].

Our study showed no significant differences in the frequencies of mtDNA haplogroups or haplogroup clusters between the eoAD patients and controls. The frequency of HV cluster was higher among the eoAD patients than among the controls, but the difference was not significant. Interestingly, this haplogroup cluster has also been reported to be significantly associated with the risk of late-onset AD independently of ApoE genotype in a Polish population [34]. In the same study, women with haplogroup H were found to have an elevated risk of AD, but the effect was no longer statistically significant after adjustment for age and ApoE genotype. In addition, a weak association has also been observed between haplogroup H and pathologically confirmed dementia with Lewy bodies [19]. In our sample the frequency of haplogroup H was slightly higher among the eoAD patients than among the controls, the relative risk (RR) being 1.28. We calculated the relative risk attached to haplogroup H among the total of 957 patients with AD in four previous association studies and the present study, involving altogether 900 controls, and found it to be 1.2 (range 1.06-1.28), which lends support to a possible role for haplogroup H regarding the risk of AD (see additional file 3).

Comparisons of nucleotide diversity and neutrality tests in European haplogroup clusters have shown that there are haplogroup-specific differences in the intensity of selection against particular regions of the mitochondrial genome, suggesting that some mutations may be non-neutral within specific phylogenetic lineages but neutral in others [35]. Haplogroup cluster HV has been shown to be most non-neutral of the European haplogroup clusters by virtue of the high number of segregating sites and singleton mutations [35]. The pathogenic potential of a mutation may depend on the presence of interacting mutations or on the presence of a certain haplogroup. It is intriguing that nonagenarians have been reported to have lower frequencies of haplogroup H and HV cluster [16] thus supporting that haplogroup H may have a role in neurodegenerative diseases and early aging.

The frequencies of the mtDNA haplogroups vary between populations and even regionally within the same country [36,37]. There are also major differences in haplogroup frequencies between northern and southern Finland [16,25], which implies that it is essential to compare the patients' data with control findings from the same area in order to obtain reliable results. Therefore, we were careful to enrol patients and controls from the same province. Furthermore, the mothers of the controls were required to have been born in the same province as the controls themselves, to ensure that they represented the ancestral population of the province. The average migration rate in the region was low during the period when the mothers of the controls must have been born [25].

There is considerable evidence of abnormal mitochondrial function and oxidative stress in AD [38,39], where impaired energy metabolism and oxidative damage may precede beta-amyloid deposition [40]. On the other hand, the level of somatic mutations is no higher in AD patients than in aged-matched controls, although mtDNA mutations do increase with age [41]. Oxidative stress and associations with mtDNA have been less intensively studied in the case of FTLD. In this investigation into the role of mtDNA and its maintenance enzymes in Finnish early-onset AD and FTLD patients the two common mtDNA mutations (m.3243A>G, m.8344A>G) and five *POLG1 *mutations were not found, nor were there any pathogenic mutations in the *PEO1 *or *ANT1 *genes. However, we cannot exclude other mitochondrial mutations as the whole mitochondrial genome was not screened.

## Conclusions

Our analysis revealed a significant association between mtDNA haplogroup cluster IWX and FTLD, suggesting that possession of this cluster may have a role in developing neurodegeneration in FTLD. Further studies in other ethnically distinct cohorts are needed to clarify the contribution of mtDNA haplogroups to FTLD and AD.

## Abbreviations

AD: Alzheimer's disease; ApoE: apolipoprotein E; ANT1: adenine nucleotide translocator; CSGE: conformation-sensitive gel electrophoresis; eoAD: early-onset Alzheimer's disease; FTLD: frontotemporal lobar degeneration; mtDNA: mitochondrial DNA; POLG1: DNA polymerase γ; PEO1: progressive external ophthalmoplegia 1.

## Competing interests

The authors declare that they have no competing interests.

## Authors' contributions

JK participated in the design of the study, carried out the molecular genetic studies, performed the statistical analysis and drafted the manuscript. RH participated in the design of the study and participated in critical revising of the manuscript. KM participated in the design of the study and participated in critical revising of the manuscript. AMR conceived of the study, and participated in its design and coordination and helped to draft the manuscript. All authors read and approved the final manuscript.

## Supplementary Material

Additional file 1 **ANT1polymorphisms among the eoAD and FTLD patients.** Table showing *ANT1 *polymorphisms detected in our cohort of the eoAD and FTLD patients Format: PDF. Size: 9.34 KB. This file can be viewed with: Adobe Acrobat Reader. Click here for file

Additional file 2 ***PEO1*****polymorphisms among the eoAD and FTLD patients.** Table showing *PEO1 *polymorphisms detected in our cohort of the eoAD and FTLD patients. Format: PDF. Size: 8.18 KB. This file can be viewed with: Adobe Acrobat Reader. Click here for file

Additional file 3 **Frequencies of mtDNA haplogroups among AD patients and controls and relative risk values calculated for the mtDNA haplogroups.** Table showing comparison of the present data with previously published findings on the frequencies of mtDNA haplogroups among AD patients and controls, and relative risk (RR) values for the mtDNA haplogroups among patients with AD. Format: PDF. Size: 26.1 KB. This file can be viewed with: Adobe Acrobat Reader. Click here for file
