# Supplementary material for: Mitochondrial DNA haplogroups in early-onset Alzheimer's disease and frontotemporal lobar degeneration
Source: Mol Neurodegener. 2010 Feb 2;5:8. doi: 10.1186/1750-1326-5-8 (PMC2830999; doi:10.1186/1750-1326-5-8)
Supplement: Additional file 1 — ANT1polymorphisms among the eoAD and FTLD patients. Table showing ANT1 polymorphisms detected in our cohort of the eoAD and FTLD patients Format: PDF. Size: 9.34 KB. This file can be viewed with: Adobe Acrobat Reader. [file 1750-1326-5-8-S1.PDF]

Additional file 1 *ANT1* polymorphisms among the eoAD and FTLD patients.

**Location**[a]	**Genome**[b]	**Predicted RNA**[c]	**Predicted protein**[d]	**rs number**	**FTLD patients** (n=66)	**eoAD patients** (n=128)	**All eoAD and FTLD patients** (n=194)
IVS1-25G>A	g.18476442G>A	c.-25G>A	-	rs3733652	4	9	13
IVS2-65_-64insT	g.18477793_18477794insT	c.112-65_112-64insT	-	rs34795113	66	128	194
**EX2+456T>C**	**g.186303368T>C**	**c.567T>C**	**p.A189A**	-	4	12	16

[a]EX=exon, IVS=intron, [b]Numbering relative to the reverse complement of GenBank accession number NT_022792.17, starting at nucleotide 1.

[c]Numbering according to GenBank accession number NM_001151.2, starting at the translation initiation codon. [d]Numbering according to the GenPept accession number NP_001142.2. The novel variant is shown in bold face.
