# Supplementary material for: Mitochondrial DNA haplogroups in early-onset Alzheimer's disease and frontotemporal lobar degeneration
Source: Mol Neurodegener. 2010 Feb 2;5:8. doi: 10.1186/1750-1326-5-8 (PMC2830999; doi:10.1186/1750-1326-5-8)
Supplement: Additional file 2 — PEO1polymorphisms among the eoAD and FTLD patients. Table showing PEO1 polymorphisms detected in our cohort of the eoAD and FTLD patients. Format: PDF. Size: 8.18 KB. This file can be viewed with: Adobe Acrobat Reader. [file 1750-1326-5-8-S2.PDF]

Additional file 2 *PEO1* polymorphisms among the eoAD and FTLD patients.

**Location**[a]	**Genome**[b]	**Predicted RNA**[c]	**Predicted protein**[d]	**rs number**	**FTLD patients** (n=66)	**eoAD patients (**n=128)	**All eoAD and FTLD patients** (n=194)
EX1+639C>T	g.21497131C>T	c.639C>T	p.G213G	rs11542130	1	0	1
EX1+1102G>A	g.21497594G>A	c.1102 G>A	p.V368I	rs17113613	7	7	14

[a]EX=exon,,[b]Numbering relative to the reverse complement of GenBank accession number NT_030059.12, starting at nucleotide 1.[c]Numbering according to GenBank accession number NM_021830.3, starting at the translation initiation codon. [d]Numbering according to the GenPept accession number NP_068602.2.
