# Supplementary material for: Mitochondrial DNA haplogroups in early-onset Alzheimer's disease and frontotemporal lobar degeneration
Source: Mol Neurodegener. 2010 Feb 2;5:8. doi: 10.1186/1750-1326-5-8 (PMC2830999; doi:10.1186/1750-1326-5-8)
Supplement: Additional file 3 — Frequencies of mtDNA haplogroups among AD patients and controls and relative risk values calculated for the mtDNA haplogroups. Table showing comparison of the present data with previously published findings on the frequencies of mtDNA haplogroups among AD patients and controls, and relative risk (RR) values for the mtDNA haplogroups among patients with AD. Format: PDF. Size: 26.1 KB. This file can be viewed with: Adobe Acrobat Reader. [file 1750-1326-5-8-S3.PDF]

Additional file 3 Comparison of the present data with previously published findings on the frequencies of mtDNA haplogroups among AD patients and controls, and relative risk (RR) values for the mtDNA haplogroups among patients with AD.

Haplogroup	Chinnery et al. 2000			Carrieri et al. 2001			Mancuso et al. 2007			Maruszak et al. 2008			Krüger et al. 2009		
	Patients	Controls		Patients	Controls		Patients	Controls		Patients	Controls		Patients	Controls	
	(%)	(%)	RR	(%)	(%)	RR	(%)	(%)	RR	(%)	(%)	RR	(%)	(%)	RR
**H**	47.6	41.3	1.15	43.2	34.1	1.27	43.8	41.4	1.06	51.4	42.8	1.20	43.8	34.3	1.28
**I**	1.6	1.7	0.94	n.d	n.d.	-	2.4	1.6	1.50	1.8	2.0	0.90	0.0	0.0	0.00
**J**	9.2	14.0	0.66	8.5	7.8	1.09	8.1	7.3	1.11	7.2	11.5	0.63	5.5	2.0	2.75
**K**	11.4	11.7	0.97	5.6	9.5	0.59	5.7	8.9	0.64	3.2	4.8	0.67	3.9	3.0	1.30
**T**	8.6	10.1	0.85	15.5	11.7	1.32	7.1	10.5	0.68	8.6	9.5	0.91	5.5	8.1	0.68
**U**	18.4	17.9	1.03	11.3	11.2	1.01	13.8	12.6	1.10	12.2	17.1	0.71	32.0	34.3	0.93
**V**	n.d.	n.d.	-	n.d.	n.d.	-	4.3	2.1	2.05	2.7	2.8	0.96	4.7	6.1	0.77
**W**	1.6	0.6	2.67	n.d.	n.d.	-	2.4	2.1	1.14	4.0	1.2	3.33	3.9	8.1	0.48
**X**	n.d.	n.d.	-	n.d.	n.d.	-	4.3	2.6	1.65	1.8	2.4	0.75	0	1.0	0.00
**Other**	1.6	2.8	0.57	16.0	25.7	0.62	8.1	10.9	0.74	7.2	5.9	1.22	0.8	3.1	0.26
**Total n**	185	179		213	179		209	191		222	252		128	99	
